# Unsupervised feature learning for electrocardiogram data using the convolutional variational autoencoder

**DOI:** 10.1371/journal.pone.0260612

**Published:** 2021-12-01

**Authors:** Jong-Hwan Jang, Tae Young Kim, Hong-Seok Lim, Dukyong Yoon

**Affiliations:** 1 Department of Biomedical Systems Informatics, Yonsei University College of Medicine, Yongin, Gyeonggi-do, Republic of Korea; 2 BUD.on Inc, Jeonju, Republic of Korea; 3 Department of Cardiology, Ajou University School of Medicine, Suwon, Gyeonggi-do, Republic of Korea; 4 Center for Digital Health, Yongin Severance Hospital, Yonsei University Health System, Yongin, Republic of Korea; National University of Sciences and Technology (NUST), PAKISTAN

## Abstract

Most existing electrocardiogram (ECG) feature extraction methods rely on rule-based approaches. It is difficult to manually define all ECG features. We propose an unsupervised feature learning method using a convolutional variational autoencoder (CVAE) that can extract ECG features with unlabeled data. We used 596,000 ECG samples from 1,278 patients archived in biosignal databases from intensive care units to train the CVAE. Three external datasets were used for feature validation using two approaches. First, we explored the features without an additional training process. Clustering, latent space exploration, and anomaly detection were conducted. We confirmed that CVAE features reflected the various types of ECG rhythms. Second, we applied CVAE features to new tasks as input data and CVAE weights to weight initialization for different models for transfer learning for the classification of 12 types of arrhythmias. The f1-score for arrhythmia classification with extreme gradient boosting was 0.86 using CVAE features only. The f1-score of the model in which weights were initialized with the CVAE encoder was 5% better than that obtained with random initialization. Unsupervised feature learning with CVAE can extract the characteristics of various types of ECGs and can be an alternative to the feature extraction method for ECGs.

## 1. Introduction

An electrocardiogram (ECG) is a record of the electrical fluctuations generated during cardiac activity. ECGs are useful for several biomedical applications, including detection of arrhythmic events, measuring heart rate variability, predicting myocardial infarctions, and screening contractile dysfunctions [1, 2]. ECGs can be collected using a wearable device, an important healthcare data can be easily obtained from ECGs for detecting and predicting the patients’ health status [3].

Many studies have attempted to develop a model that predicts a patient’s disease or health state via electrocardiography; however, feature extraction is required to reduce the high-dimensional data included in ECGs. Various feature extraction methods have been proposed for ECG analysis, including morphological and temporal approaches. In the morphological approach, the specific shape of an ECG waveform, such as ST elevation, ST depression, and J-waves, is defined [4, 5]. In the temporal approach, the time interval between certain features observed in ECGs, such as the PR, RR, and QT intervals, is defined [6, 7].

However, the various shapes of the waveforms of ECGs render rule-based feature extraction challenging. A morphological feature must be defined to be extracted; however, it is impossible for a researcher to screen and define all waveform shapes observed in ECGs. Similarly, temporal features can only be defined by intervals between certain features that can be recognized.

Deep learning was recently proposed for feature extraction of high-dimensional data. There are two approaches to deep learning: supervised and unsupervised learning. In supervised learning, feature extraction is performed to classify data according to a matched label. Moreover, supervised learning has recently been applied to ECG analysis. Zhu et al. [8] conducted a study to classify arrhythmias using a convolutional neural network (CNN), while Attia [9] showed that deep learning could predict potential arrhythmic events using normal sinus rhythms (SRs) only. These results indicate that deep learning can recognize and capture features that are difficult to be perceived with the existing rules. In unsupervised learning, feature extraction is performed with unlabeled data only. Chiang et al. [10] conducted a study on restoring a noisy ECG using a denoising autoencoder. However, in supervised feature learning, each ECG feature must be labeled, thus making the process time-consuming and resource-intensive. In addition, the application of features extracted by supervised learning to other tasks is difficult, as feature learning focuses on a specific task.

To overcome the limitations of supervised feature learning and represent the general features of ECGs, unsupervised learning methods including the use of an autoencoder, were applied for the analysis of ECGs. However, most of the unsupervised feature learning methods were applied only to ECG beat units, while the clinically relevant rhythm of ECGs is a combination of several beats. Therefore, studies on beat units cannot accurately reflect the ECG characteristics. Even in studies conducted using ECG rhythms, either the scale of the ECG dataset or the performance evaluation was not adequate, and external validation was not performed.

This study proposed an unsupervised ECG feature learning method, using the convolutional variational autoencoder (CVAE), and determined the effectiveness of the features. We trained CVAE using data from the Ajou University Medical Center Intensive Care Unit (AUMC) biosignal database to include the ECG features of various types of patients. Validation of CVAE features was then conducted via two approaches with external datasets. The first validation method was to analyze the CVAE features without an additional training process using clustering, latent exploration, and anomaly detection. Next, we applied trained CVAE and CVAE features to a new task for training a different machine learning model to validate the CVAE. This included two types of transfer learning for the classification of 11 ECG rhythms. We hypothesized that the CVAE can extract features reflecting characteristics of ECGs without any labeled ECG data.

Although only lead II ECG of the intensive care unit (ICU) was utilized, no other study has validated unsupervised feature learning trained with ECGs from large-scale ICU and provided its model and weights. We have contributed to not only the application and validation of unsupervised feature learning through anomaly detection, clustering, latent space exploration, and transfer learning, but also the provision of pretrained model weights applicable to research.

## 2. Materials and methods

This study was approved by the Ajou University Hospital Institutional Review Board (AJIRB-MED-MDB-20-302). The requirement of obtaining informed consent was waived owing to the retrospective nature of this study.

### 2.1 Data sources

The AUMC ICU biosignal database [11, 12] was collected from 23,422 patients in the ICU at Ajou University Hospital between 2017 and June 1, 2020. The AUMC ICU biosignal database includes two types of biosignal data, namely, waveform data such as ECG data, arterial blood pressure, photoplethysmogram data, and respiratory impedance using the Nihon Kohden and Philips device, and numeric data including the heart rate and respiratory rate, etc. In this study, the CVAE was trained using only ECG lead II data, which were collected at 250 or 500 Hz, when using a Philips or Nihon Kohden device, respectively. We randomly selected 1,300 patients and extracted ECG samples of 8.2 seconds in length at 10-minute intervals throughout each patient’s admission. The AUMC dataset was divided into a training dataset and a validation dataset at a ratio of 8:2.

We also used a 12-lead ECG dataset from the Shaoxing People’s Hospital in China, which includes 12-lead ECG data recorded at 10-second intervals using a GE MUSE ECG system at 500 Hz [13]. This dataset includes ECGs from 10,646 patients. Each ECG was categorized by two physicians into one of 11 rhythm types: normal sinus rhythm (SR), sinus irregularity (SI), sinus bradycardia (SB), sinus tachycardia (ST), supraventricular tachycardia (SVT), atrial tachycardia, sinus atrium to atrial wandering rhythm, atrioventricular node reentrant tachycardia, atrioventricular reentrant tachycardia, atrial fibrillation (AFIB), and atrial flutter. The categories were crosschecked by two physicians. Data from lead II were used for clustering, anomaly detection, and transfer learning for the 11-rhythm classification task.

The third dataset was the Massachusetts institute of technology-Beth Israel hospital (MIT-BIH) biosignal dataset from Beth Israel Hospital in Boston, USA [14, 15]. We used the MIT-BIH arrhythmia dataset collected from 47 patients diagnosed with arrhythmias as well as the normal SR dataset collected from 18 patients who had no significant arrhythmias. ECG lead II were used to externally validate the anomaly detection threshold generated using the Shaoxing dataset. A total of 542 samples of arrhythmias were extracted from the MIT-BIH arrhythmia dataset and 30 samples of normal ECGs were extracted for each patient from the MIT-BIH normal SR dataset.

The fourth dataset was a 12-lead ECG dataset from a medical check-up at AUMC. AUMC check-up dataset had been collected by GE MUSE system from July 1994 to September 2012. A total of 246,266 ECG test data were stored. We extracted 1,000 samples to resemble the age distribution of AUMC ICU dataset and utilized them for clustering and visualizing ECG feature of ICU and medical check-up population.

### 2.2 Data preprocessing

We removed baseline noise < 0.5 Hz using a Butterworth pass filter. Data were resampled to 250 Hz, and linear interpolation was applied when up-sampling was required. After resampling, the ECG data were normalized via z-score normalization. The lengths of the data were adjusted to 8.2 seconds so that each data contained a total of 2,048 values.

### 2.3 Model development

#### 2.3.1 One-dimensional convolutional variational autoencoder

**Fig 1:**
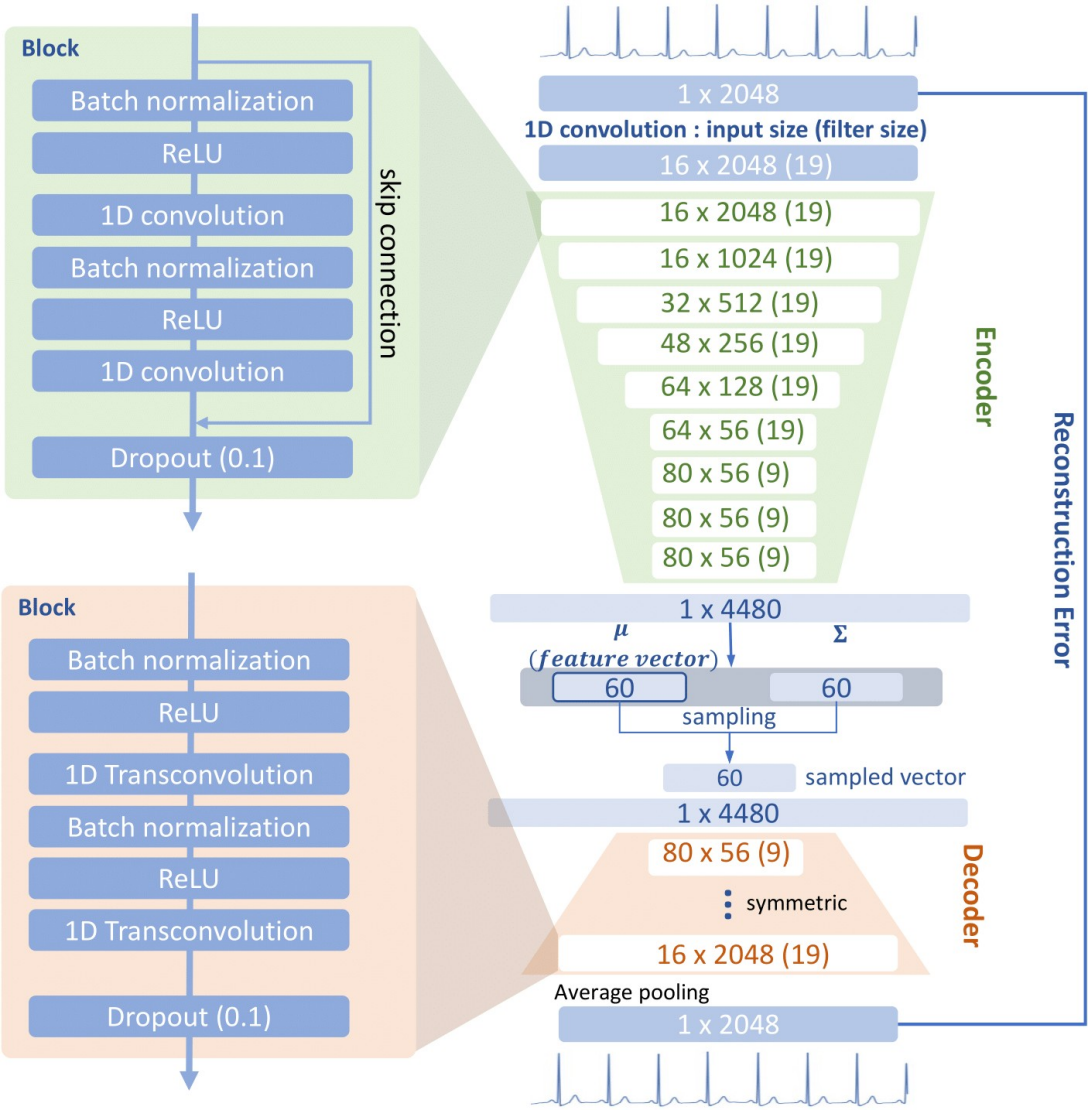
Model architecture of encoder in convolutional variational autoencoder (CVAE) consists of nine layers of one-dimensional convolutional neural network (CNN). The filter size is 19 until the sixth residual block, after which it is reduced to nine. At the end of encoder, the extracted data are flattened and converted into the values of mean and standard deviation with a size of 60. The latent variable is sampled from the values of mean and standard deviation. The hyperparameter of the decoder is set symmetrically so that the output size is the same as that of the encoder. Average pooling is applied to the outputs of the decoder. The mean values were used as the CVAE feature vector of electrocardiogram (ECG).

The CVAE consisted of an encoder and a decoder [16]. The encoder consisted of one-dimensional convolutional neural networks (1D CNNs) used to extract latent variables smaller than the original ECG data (Fig 1). The decoder consisted of transposed 1D CNNs trained to reconstruct output similar to the original ECG data using latent variables from the encoder. When the encoder extracted latent variables, it generated means and standard deviations and then, generated latent variables sampled from a multivariate Gaussian distribution based on the means and the standard deviations. In this study, mean values were used as CVAE features, the size of the mean values was set at 60. The CVAE was trained to minimize the reconstruction error between the original ECG and reconstructed ECG and to restrict means and standard deviations close to zeros and ones. By restricting means and standard deviations, we were able to estimate the distribution of CVAE features. More detailed structures of the CVAE model are described in S1 Appendix.

### 2.4 Evaluation

#### 2.4.1 Evaluation of CVAE

The CVAE performance was evaluated using the sum of the reconstruction error and Kull-back divergence. Smaller values indicate a better performance. During variational autoencoder training, the model performance was evaluated through a validation dataset for each epoch, and early stopping was performed when there was no improvement in performance during an epoch.

#### 2.4.2 Evaluation of classification task

Two classification tasks—anomaly detection and transfer learning (classifying ECGs into 11 ECG rhythms)–were evaluated using four evaluation metrics: accuracy, precision, recall, and weighted f1-score. The area under the receiver operating characteristic (AUROC) [17] is also presented in the evaluation of anomaly detection. To confirm the difference in two distributions, a two-sided Welch’s t-test was used with the significance level set at 0.05.


Accuracy=(TruePositive+TrueNegative)/(Totalnumberofdata)

Precision=(TruePositive)/(TruePositive+FalsePositive)

Recall=(TruePositive)/(TruePositive+Falsenegative)

f1−score=2*(Presion*Recall)/(Precision+Recall)

$$weighted\;f1-score=\sum_{i=1}^{k}w_{i}*{f1-score}_{i}$$

$$w_{i\;}:\;portion\;of\;class\;i\;in\;total\;classes,\;k:\;the\;number\;of\;classes$$

### 2.5 Validation of CVAE features

To validate CVAE features, anomaly detection, features clustering, latent space exploration and transfer learning were conducted.

#### 2.5.1 Anomaly detection

The reconstruction error of CVAE was used as an anomality feature. As the reconstruction error fully depends on the feature vector, the anomality score can be considered a CVAE feature. The larger the anomality score, the higher the probability of anomalous ECG. In the expanded methods in S2 Appendix, the assumptions for anomaly detection with the reconstruction error are described in detail. We conducted anomaly detection to determine the effectiveness of the anomality feature using the Shaoxing dataset. In this process, SR, SI, SB, and ST were defined as normal ECGs, and the remaining rhythms were defined as abnormal ECGs. Moreover, we conducted an additional anomaly detection experiment to compare with previous work: long short-term memory (LSTM)-VAE. More detailed methods are described in the S3 Appendix.

SI was regarded as normal data according to origin paper for Shaoxing dataset. Sinus bradycardia was included as normal data as this rhythm can occur in healthy patients without adverse effects and does not require urgent treatment. Similarly, sinus tachycardia is also typically not a serious heart rhythm. Moreover, sinus bradycardia and sinus tachycardia rhythms retain the morphological characteristics of a normal SR. The anomality score was calculated for each datum. The threshold of score for anomaly detection was selected at the highest f1-score in the Shaoxing dataset while changing the threshold by 0.05. Anomaly detection was evaluated by f1-score, precision, recall, accuracy, and the AUROC, which were described in the evaluation session in detail. The threshold of the anomality score selected by the Shaoxing dataset was applied to the MIT-BIH dataset. Moreover, we selected 1,000 patients from the AUMC ICU database and applied anomaly screening to the ECGs obtained every 10 minutes during their entire hospital admissions. We then used a histogram to visualize the anomality score distribution.

#### 2.5.2 Clustering feature vectors

Clustering was performed using t-stochastic neighbor embedding (T-SNE) [18] and locally linear embedding (LLE) [19]algorithms to confirm whether the CVAE features reflect characteristics of various ECG rhythms. Three hundred samples were randomly selected from the Shaoxing dataset for each rhythm category. Clustering was conducted with normal sinus samples and each of the arrhythmia samples. To determine whether distribution of ECG in ICU differ with distribution of ECG in Non-ICU population, we conducted clustering with 1,000 ECG samples from ICU and from medical check-up, respectively.

#### 2.5.3 Latent space exploration

Latent space exploration allows for the identification of the indirect implications of the feature vector [20]. While the value of the feature vectors was changed, we visualized whether reconstruction output. After adding or subtracting 2 to a certain value in the feature vector, we observed the result of the reconstruction output. If the CVAE learned features according to the various characteristics of ECGs, reasonable changes are expected, in line with the ECG domain.

#### 2.5.4 Transfer learning for arrhythmia classification

We conducted two types of transfer learning. First, we reused the extracted features from CVAE as the input data for a different model. Second, we reused the weights of the trained CVAE encoder to initialize another model. The usefulness of the CVAE features and weights were confirmed by applying them to the task of categorizing ECGs from the Shaoxing dataset into 11 categories of ECG rhythms. We used an 8:2 ratio of training and validation data and repeated the validation model five times using the bootstrap method [21]. The classification results were shown as the mean and standard deviation of each metric. For the feature reuse experiment, we conducted arrhythmia classification using the extreme gradient boosting (XGBoost) model [22] with CVAE features as input data. Moreover, to conform that the anomality score offers additional information, we classified the arrhythmias using only features of CVAE as well as features of CVAE and anomality features.

We compared the performance of the model applied with CVAE weights and the model applied with randomly initialized weights. The initial weight setting is known to affect performance and convergence speed. If the CVAE encoder is trained to learn meaningful feature extraction from ECGs, the classification will be affected by the weight settings. The performance and convergence speed of transfer learning was compared with a randomly initialized model. In this study, the classification model has an encoder and a fully connected layer. The detailed structure of this model is described in S1 Fig. The learning rate was 0.0001 and the total number of epochs was 150.

### 2.6 Software

CVAE model construction was performed on the framework with Pytorh 1.3 in Python 3.6 (Python Software Foundation, Wilmington, DE, USA). Model evaluation was conducted on the platform with sklearn 0.21. The XGBoost package 1.0.2 was applied to the XGBoost model for transfer learning.

## 3. Results

### 3.1 Patient and dataset characteristics

Table 1 summarizes the patients’ demographic data and the ECG data information for the four datasets used in this study. The AUMC ICU dataset included 1,278 patients for whom demographic information could be obtained. There were no significant differences in the mean age of the patients in each dataset. The AUMC ICU dataset contained significantly more male patients than the other three datasets (P-value<0.05). Half (50.1%; 540/1,082) of the ECGs in the MIT-BIH dataset and 71.2% (7,682 /10,646) in the Shaoxing dataset were considered normal, as defined in the anomaly detection methods section. In the anomaly detection experiments, ST and SB were regarded as normal ECG because there was no morphological difference compared with normal sinus rhythm and these can be easily detected by heart rate.

**Table 1:** Baseline characteristics of each dataset.

Characteristic		Datasets			
		AUMC ^a^ ICU dataset (n = 1,278)	Shaoxing dataset (n = 10,646)	MIT-BIH^b^ dataset (n = 65)	AUMC Check-up dataset (n = 1,000)
Age (year), Mean (±SD)^c^		60.23 (19.17)	59.2 (18.00)	53.71 (22.4)	60.98 (14.05)
Men, No. (%)^d^		892 (64.68)	5,956 (52.08)	31 (47.70)	506 (50.06)
ECG data for study (N)		596,547	10,646	1,082	1,000
	ECGs per patient,	466.82	1	16.61	1
Anomaly detection	Normal, No.	-	7,682	540	-
	Arrhythmia, No.	-	2,964	542	-

^a^Ajou university medical center.

^b^Massachusetts institute of technology-Beth Israel hospital.

^c^Continuous variables are shown as mean (±SD).

^d^Categorical variables are shown as n or n (%).

### 3.2 CVAE training results

The CVAE was trained to extract latent variables that follow multivariate normal Gaussian distributions *N*(*I*,0) and then reconstruct the original ECG data using these variables. CVAE performance was evaluated using the sum of the reconstruction error and Kull-back divergence. The reconstruction error represents how well the CVAE extract ECG information, and Kull-back divergence represents how well the distribution of the latent variables follows a multivariate normal Gaussian distribution. When the sum of the reconstruction error and KL-divergence was not decreased over 5 epochs, the training of the CVAE was stopped early, at the 18^th^ epoch. The reconstruction error was 560.31 and the KL-divergence was 79.72. The mean error per datum point was 0.27 (560.31/2,048). As the average y-axis range of zero-normalized ECG data is from -2 to 6, each value had an error of 3.38%.

### 3.3 Clustering feature vectors

**Fig 2:**
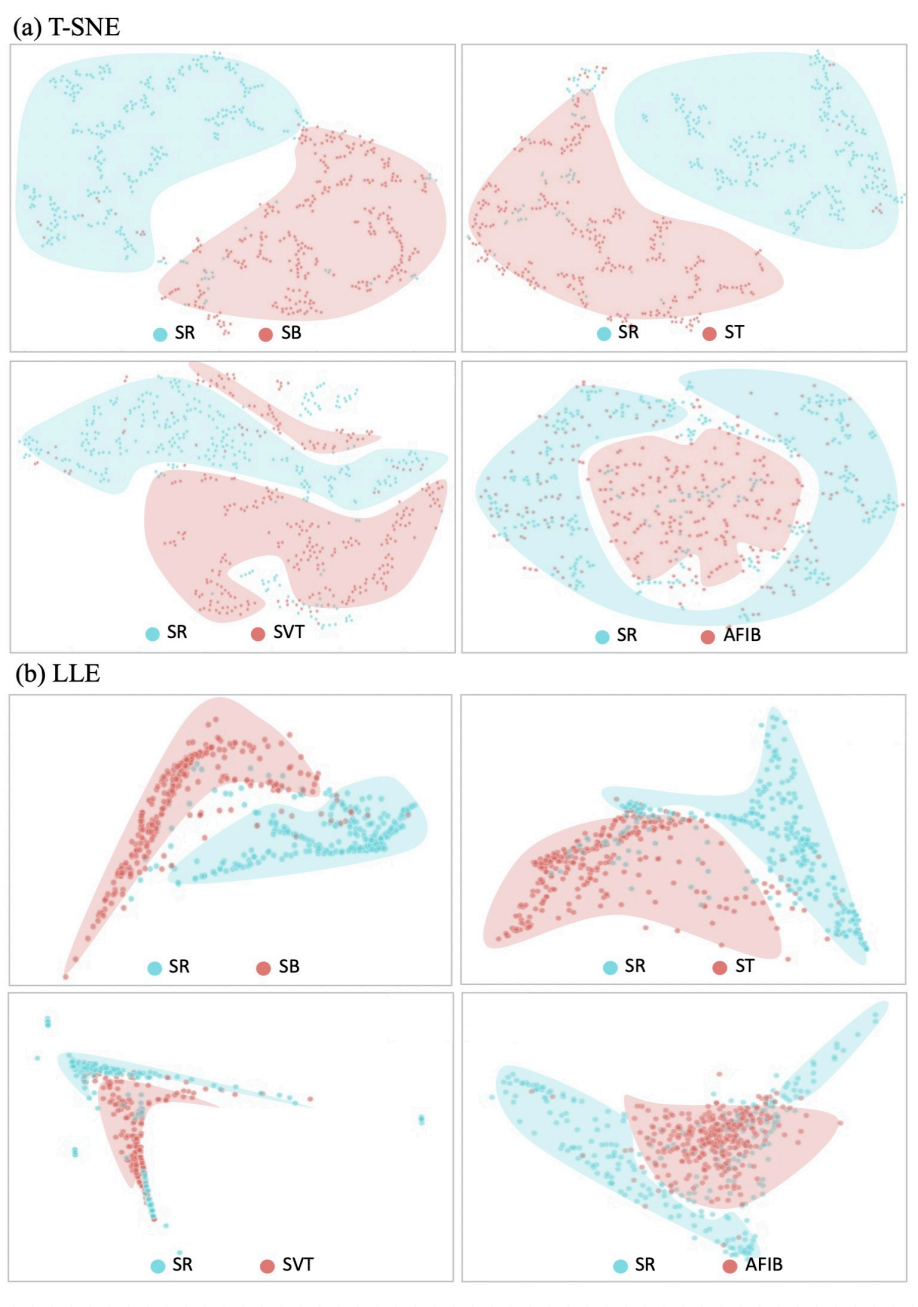
Visualization of (a) t-Stochastic Neighbor Embedding (t-SNE) and (b) Locally Linear Embedding (LLE) in two-dimensional space for normal sinus rhythms and arrhythmias. Normal sinus rhythms are shown as blue clusters while arrhythmias are shown as red clusters. It shows that feature vectors can represent the differences between normal and arrhythmic rhythms although the model was trained without any label. Abbreviations: SR: normal sinus rhythm; ST: sinus tachycardia; SVT: supraventricular tachycardia; AFIB: atrial fibrillation.

**Fig 3:**
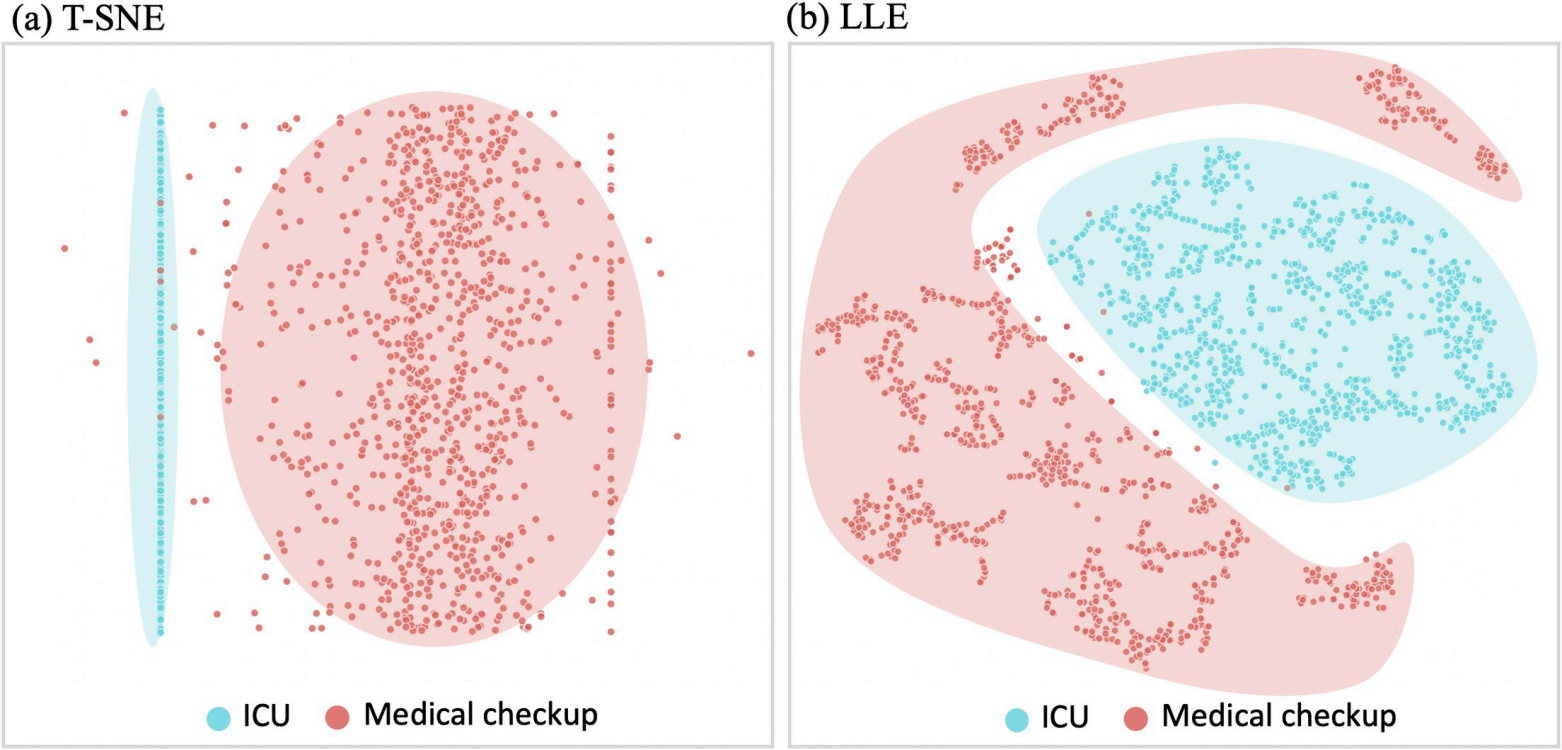
Visualization of (a) t-SNE and (b) LLE in two-dimensional space for ECG from ICU and Healthy test. ECGs from ICU are shown as blue clusters while ECGs from medical-checkup are shown as red clusters. It shows that feature vectors can represent the differences between healthy and unhealthy people although the model was trained without any label.

The features learned by the CVAE were visualized in a two-dimensional space using t-Stochastic Neighbor Embedding (t-SNE) (Fig 2A). The features of each arrhythmia were clustered with the normal SRs. Although CVAE was not trained to classify arrhythmias, the features show that it implied and learned the various patterns of arrhythmias. The T-SNE clustering method showed that there were no cluster groups of AFIB, however, the LLE method showed features that were divided into an AFIB group and a normal group (Fig 2B). The results of clustering for arrhythmias with less than 300 samples are shown in S2 Fig. Fig 3 shows that features from ICU and from medical checkup are divided into two clustered groups. Both results of clustering showed distinct feature distribution between ICU and medical check-up population.

### 3.4 Reconstruction output validation (latent space exploration)

**Fig 4:**
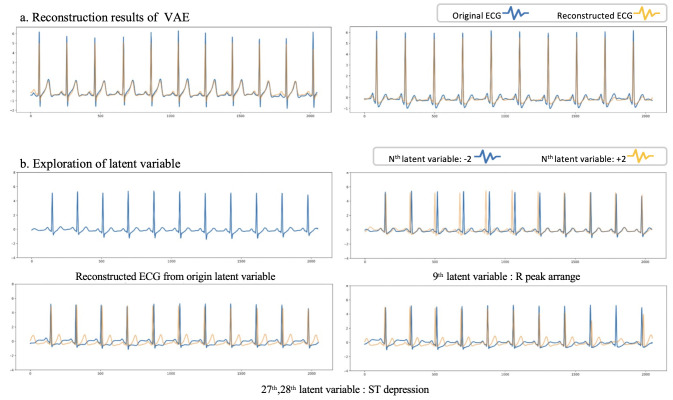
Reconstruction output and feature space exploration. **(a)** Examples of the reconstruction output of a normal electrocardiogram (ECG) are shown. Original ECG data (blue) and reconstructed ECG data (yellow) are not significantly different. **(b)** Examples of latent space exploration. A single blue line represents the reconstruction output of the sampled origin feature vector in the first ECG. In the remaining three ECGs, the blue line represents the reconstruction output of the feature vector that subtracts 2 from the Nth feature variable. The yellow lines represent the reconstruction output of the feature vector that adds 2 to the Nth feature variable.

To validate the feature vector space of CVAE and verify that the CVAE learned to imply clinically meaningful changes, we changed each variable in a feature vector and observed the reconstruction output of the changed feature vector. Fig 4A shows an example of a reconstruction of a normal SR. It is confirmed that the reconstruction outputs are close to the original data. In Fig 4B, the reconstruction output resulting from a changed latent variable shows clinically meaningful changes. The 9^th^ latent variable affects the R peaks and the 27^th^ and 28^th^ latent variable affect the ST depression. These changes to the ECGs are significant and provide validation for the meaningful latent space learned by the CVAE.

### 3.5 Anomaly detection

We detected anomalies by defining a normal ECG as an ECG rhythm with no evidence of arrhythmia. In the anomaly detection experiments, SB and ST were included in the normal ECG group. We defined the reconstruction error of CVAE as an anomality score. We performed anomaly detection to determine the effectiveness of the anomality score using the Shaoxing and MIT-BIH datasets. The threshold of anomalous ECG was determined by the Shaoxing dataset and applied to the MIT-BIH dataset. Table 2 shows the performance of the anomaly detection through the reconstruction error of the CVAE. The performance of anomaly detection showed the highest f1-score of 0.82 when the threshold was set to 0.45 in the Shaoxing dataset. When a threshold of 0.45 was applied to the MIT-BIH dataset, the f1-score, precision, and recall of abnormal ECG were 0.72, 0.88, and 0.53, respectively. The AUROC was 0.85 in the Shaoxing dataset and 0.84 in the MIT-BIT dataset. The distribution of the anomality score between normal and abnormal ECGs was significantly different (P-value<0.05). Moreover, we conducted additional anomaly detection using Long Short-Term Memory (LSTM)-VAE for comparison with recent work [23]. The LSTM-VAE results are described in the S3 Appendix.

**Table 2:** Anomaly detection in the Shaoxing and MIT-BIH datasets.

Dataset	Threshold	Weighted f1-score	Accuracy	Normal		Abnormal	
				Precision	Recall	Precision	Recall
**Shaoxing**	0.25	0.69	0.67	0.93	0.59	0.45	0.89
	0.35	0.80	0.80	0.89	0.83	0.62	0.73
	0.45 ^a^	0.82	0.83	0.85	0.92	0.75	0.58
	0.55	0.73	0.82	0.82	0.97	0.83	0.44
**MIT-BIH**	0.45	0.72	0.73	0.66	0.92	0.88	0.53

^a^The threshold with the highest micro f1-score was selected as the criteria for abnormal electrocardiograms. When thresholds with the same f1-score were present, the threshold with the highest precision was selected.

### 3.6 Distribution of anomality score in whole electrocardiograms

**Fig 5:**
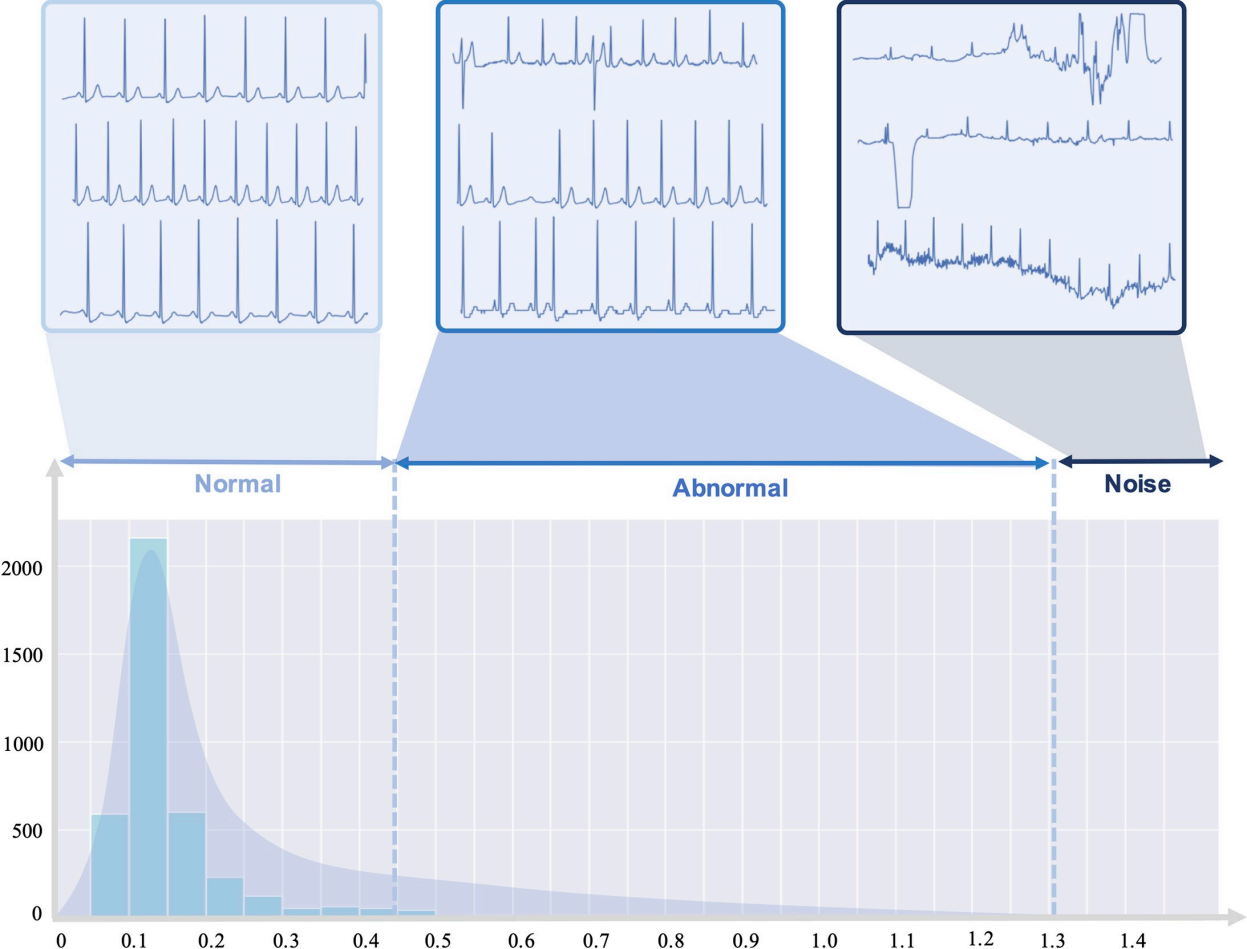
Distribution of anomality score in 1,000 patients. The distribution of the anomality score is skewed to the left, which is consistent with the premise that the number of normal ECGs is more than the number of abnormal ECGs. ECGs with an anomality score over 1.3 were due to noise or movement artifacts. The majority of ECGs with an anomality score between 0.45–1.3 were abnormal.

Fig 5 shows the results of the anomaly features obtained from the entire hospital stay of 200 patients who were randomly selected from the AUMC ICU biosignal database. The distribution of the anomality score is skewed to the left, which is consistent with the premise that the number of normal ECGs is more than the number of abnormal ECGs. ECGs with an anomality score over 1.3 were due to noise or movement artifacts. The majority of ECGs with an anomality score between 0.45–1.3 were abnormal.

### 3.7 Transfer learning

We evaluated the effectiveness of the CVAE encoder and features via two types of transfer learning, which is a method where a trained model is reused for a different model [24]. One type is feature reuse where features of CVAE were applied as an input for a different model. In this study, CVAE features were used as an input for the XGBoost model for classifying cardiac rhythms. The other type is weight reuse where weights of the CVAE encoder was used to initialize another model. Classification task with transfer learning was conducted with the Shaoxing dataset including 11 cardiac rhythms.

The accuracy, f1-score, precision, and recall of the CVAE are 0.83, 0.86, 0.89, and 0.83, respectively, when transfer learning of feature reuse for classification 11 cardiac rhythms was performed using only CVAE features (Table 3). The overall performance of classification task improved when using the CVAE features and anomality features. The results of each arrhythmia and feature importance are described in S1 and S2 Tables, respectively.

**Table 3:** Transfer learning using CVAE features and anomality features.

	Validation result of bootstrap						
		CVAE features			CVAE + anomality features		
Rhythm type	N	f1-score Mean (^a^±SD)	Precision Mean (±SD)	Recall Mean (±SD)	f1-score Mean (±SD)	Precision Mean (±SD)	Recall Mean (±SD)
All types	1,834	0.86 (0.01)	0.89 (0.00)	0.83 (0.01)	0.86 (0.01)	0.90 (0.00)	0.84 (0.01)
SB	778	0.96 (0.00)	0.97 (0.00)	0.94 (0.00)	0.96 (0.01)	0.97 (0.01)	0.94 (0.01)
SR	370	0.86 (0.02)	0.91 (0.04)	0.81 (0.01)	0.87 (0.02)	0.91 (0.05)	0.84 (0.01)
AFIB	357	0.74 (0.02)	0.82 (0.02)	0.67 (0.02)	0.76 (0.01)	0.87 (0.01)	0.68 (0.02)
ST	329	0.86 (0.02)	0.87 (0.04)	0.81 (0.01)	0.87 (0.01)	0.88 (0.02)	0.87 (0.01)

CVAE: convolutional variational autoencoder; SB: sinus bradycardia; SR: sinus rhythm; AFIB: atrial fibrillation; ST: sinus tachycardia.

To verify that the CVAE encoder learned useful features from the ECG data, we reused the weights of the encoder to classify 11 types of ECG rhythms. The detailed results of this classification are described in Table 4. The average f1-scores for the bootstrap method of the four most common rhythms are the same for random initialization and transfer learning. In SR and AFIB rhythms, transfer learning showed a better performance and more stable results than random initialization.

**Table 4:** Transfer learning vs. random initialization using the bootstrap validation method.

	Validation result of bootstrap						
		Random initialization^a^			Transfer learning^b^^,^^c^		
Rhythm type	N	f1-score Mean (^a^±SD)	Precision Mean (±SD)	Recall Mean (±SD)	f1-score Mean (±SD)	Precision Mean (±SD)	Recall Mean (±SD)
All types	1,834	0.79 (0.14)	0.78 (0.15)	0.81 (0.12)	0.79 (0.02)	0.82 (0.02)	0.80 (0.03)
SB	778	0.97 (0.00)	0.96 (0.01)	0.99 (0.00)	0.93 (0.01)	0.96 (0.04)	0.92 (0.06)
SR	370	0.71 (0.40)	0.71 (0.40)	0.72 (0.40)	0.84 (0.04)	0.85 (0.05)	0.85 (0.09)
AFIB	357	0.53 (0.48)	0.51 (0.47)	0.55 (0.50)	0.71 (0.18)	0.78 (0.05)	0.70 (0.26)
ST	329	0.94 (0.01)	0.94 (0.02)	0.93 (0.02)	0.91 (0.02)	0.92 (0.06)	0.90 (0.04)

^a^Result at the 149^th^ epoch, where the model showed the best weighted f1-score for 150 epochs.

^b^Result at the 140^th^ epoch, where the model showed the best weighted f1-score for 150 epochs.

^c^ Weights of CVAE encoder were reused for weight initialization for a classification model.

CVAE: convolutional variational autoencoder; SB: sinus bradycardia; SR: sinus rhythm; AFIB: atrial fibrillation; ST: sinus tachycardia.

The weighted average of the metrics for validation dataset for each epoch during 150 epochs is shown in S3 Fig. It shows that transfer learning reached the higher peak earlier than random initialization for all f1-scores and recall metrics. In the precision metric, random initialization reached a peak earlier, but had worse results than transfer learning. The final performance of transfer learning was more stable than the final performance of random initialization. The results of transfer learning for arrhythmias less than 300 samples are shown in S3 Table.

## 4. Discussion

The present study reports the success of unsupervised ECG feature learning using the CVAE. The codes and weights of the CVAE used in the study are available (see the Code Availability section) and can be used by researchers aiming to improve automated ECG analyses. To the best of our knowledge, this is the first study to use data from a large ICU ECG database for unsupervised feature learning. Although previous studies have used autoencoder-based feature learning, feature validation included clustering or classification tasks only. We applied and suggested four types of evaluation methods, including clustering, classification, latent space exploration, and transfer learning. By providing various application results, we confirmed the reliability and utility of unsupervised feature learning. Moreover, due to the nature of data-driven deep learning, the more the collected data, the more the information the CVAE will represent as a useful feature extractor.

In this study, the reconstruction error of the CVAE could be used as a feature to reflect anomality. The AUROCs for anomaly detection were 0.85 and 0.84 for the Shaoxing and MIT-BIH datasets, respectively. The anomality score threshold of 0.45 showed a precision of 0.75 and 0.88 for identifying abnormal ECGs in the Shaoxing and MIT-BIH datasets, respectively. However, the recall showed relatively unfavorable performances of 0.58 and 0.73 for the Shaoxing and MIT-BIH datasets, respectively. In the intensive care unit where many alarms occur sporadically, the alarm fatigue of the clinical staff is a major concern. Therefore, the selection of a threshold with a higher precision than recall is more practical for reliable anomaly detection. Consequently, some abnormal ECGs may be overlooked. A false negative analysis was conducted on 1,237 samples in the Shaoxing dataset. AFIB ECGs accounted for 56% (n = 692) of false negative samples. We compared the portion of beats that were 50% longer or shorter than the previous beat of true positive AFIB ECGs and false negative AFIB ECGs. The portion of true positive AFIB ECGs was significantly larger than false negative AFIB ECGs (P-value<0.05). This indicates that true positive samples may be more severe, which can be measured by the anomality score of ECGs. Furthermore, when screening the ECGs from the entire hospitalizations of 1,000 patients selected randomly from the AUMC ICU database, we found that noisy ECG can be classified.

The CVAE features accurately reflect clinically-relevant morphological features, such as changes in RR intervals or ST depression, as verified by the latent space exploration. However, each feature value does not correspond to a specific clinical change. In future studies, the relationship between the CVAE feature values and clinical conditions should be investigated for interpreting each feature. If the clinical relationship with the extracted features can be interpreted, the black box problem of deep learning can be alleviated, and the clinical utility can be increased. Furthermore, by utilizing the characteristics of VAE latent space exploration to make various changes, we expect to use it for data augmentation when data are insufficient.

Although the CVAE learned ECG features without any labels for arrhythmia, the CVAE features of arrhythmia were confirmed to have different distributions than those of normal sinus, as confirmed using two different clustering algorithms. Although AFIB ECGs had no clustered groups when T-SNE was applied, the LLE algorithm clustered AFIB ECGs. It visually shows that CVAE features imply information about the arrhythmia waveform. Moreover, we found that ECG features of ICU patients have different distributions compared to those of the medical check-up population. Although VAE was trained with unlabeled AUMC ICU dataset, it can represent various ECGs according to the health state: non-healthy and healthy.

We also validated the CVAE using transfer learning for the classification of ECGs into arrhythmia categories using XGBoost. The accuracy and f1-score were 0.84 and 0.86 when using a combination of CVAE and anomality features, indicating that the CVAE features are able to classify various ECG rhythms even though the CVAE was trained with unlabeled ECG data. Although the performance was not significantly different between the model using only CVAE features and the model using CVAE and anomality features, the anomality feature provided additional information, as the anomality score was determined to be the most important feature. Weight initialization with CVAE features shows that the fluctuation of evaluation metrics from transfer learning is more stable and reaches the peaks earlier than random initialization, indicating that the CVAE encoder effectively learned the weights for extracting the important features of ECG.

This study is not without limitations. Although we included a large sample size, the CVAE was developed using data from a cohort of patients from the AUMC ICU. However, there are currently few hospitals worldwide that collect such vast amounts of ECG data in the ICUs. It is expected that the performance of the model can be improved by learning various ECG data collected in multiple regions and countries in the future. The number of patients in the AUMC ICU dataset is less than that in the Shaoxing dataset. However, the number of ECG samples in the AUMC ICU dataset is five times the number of samples in the Shaoxing dataset. Moreover, the CVAE can learn internal variability by extracting various ECG samples from a patient. In this study, the length of ECG was static at 8.2 seconds because it depended on model structure and only ECG lead II was trained because most ICUs mainly monitored and collected ECG lead II. Therefore, various models that can extract features from different lengths and types of ECG should be developed in the future. Although the proposed threshold for anomaly detection may not be applicable in all circumstances, it can be used for reference when choosing a threshold for a specific cohort. Some people in the AUMC check-up dataset can be unhealthy. However, it is obvious that people undergoing medical check-ups are relatively healthier than ICU patients. In addition, the clustering results can support its distinct distribution.

## 5. Conclusion

In this study, we showed that unsupervised feature learning could be applied to ECG data using a CVAE that can learn various ECG features. Despite the absence of labels, we confirmed that unsupervised feature extractor could be trained, and its features could imply and represent various arrhythmic ECGs. Our findings can be applied by researchers and physicians in their laboratories and clinics, and a more advanced model can be developed. In the future, we plan to study the interpretation of CVAE features to determine specific clinical relevance in future studies.

## Supporting information

S1 Appendix Model development of one-dimensional convolutional variational autoencoder (CVAE). (PDF)

S2 Appendix Assumption of anomaly detection. (PDF)

S3 Appendix Anomaly detection comparison with long short-term memory (LSTM) Variational autoencoder (VAE). (PDF)

S1 Fig Visualization of T-SNE and LLE clustering. (PDF)

S2 Fig Performance of the classification of electrocardiograms into 11 rhythms in a validation dataset for each epoch. (PDF)

S3 Fig Structure of transfer learning of weight initialization. (PDF)

S1 Table Detailed results of classification of 11 rhythms using XGBoost. (PDF)

S2 Table Feature importance of XGBoost. (PDF)

S3 Table Classification of arrhythmias in the Shaoxing dataset using weight initialization of transfer learning. (PDF)
