# Supplementary material for: Unsupervised feature learning for electrocardiogram data using the convolutional variational autoencoder
Source: PLoS One. 2021 Dec 1;16(12):e0260612. doi: 10.1371/journal.pone.0260612 (PMC8635334; doi:10.1371/journal.pone.0260612)
Supplement: S1 Appendix — (PDF) [file pone.0260612.s001.pdf]

S1 Appendix. Model development of one-dimensional convolutional variational autoencoder (CVAE)

Convolutional neural network (CNN) is a deep learning model used mainly for image analysis. While scanning data through a filter, the morphological characteristics of the data are extracted. Autoencoder is an unsupervised deep learning method that can compress data into smaller features. Autoencoder consists of an encoder that compresses the original data (X) into a small, latent variable (Z) as a feature vector and a decoder that restores values close to the original data using latent variable Z. The autoencoder is trained to minimize reconstruction error, defined as the difference between the original data and the restored data. When the autoencoder is able to restore the original data with latent variables, it is assumed that the feature vector contains the essential features of the original data.

The variational autoencoder is a probability model modified from the autoencoder that learns the probability distribution of the latent variable Z. A variational autoencoder also consists of an encoder and a decoder, but the encoder generates the means and standard deviations. The mean values were used for the CVAE feature vector of the ECG data. The latent variable Z was sampled from a multivariate Gaussian distribution that follows the means and standard deviations generated from the encoder. Therefore, the variational autoencoder is trained with the goal of minimizing reconstruction error and Kull-back divergence in order to reinforce multivariate Gaussian distribution to have a mean of zero and a standard deviation of one. As a result, the latent variable Z is constrained to follow multivariate Gaussian distribution and has the advantage that the data representation is done in a certain space.

$$\mu, \Sigma = f_{encoder}(X)$$

$$Z = sample(\mu, \Sigma)$$

$$X' = f_{decoder}(Z)$$

$$Loss = Reconstruction\ Error(X, X') + D_{kl}(N(\mu, \Sigma)\ ||\ N(0, I))$$

$$X : original\ data \quad X' : reconstructed\ data \quad Z : latent\ variable$$

$$\mu, \Sigma : means\ and\ standard\ deviations\ which\ Z\ follows$$

$$Reconstruction\ error : Mean\ squared\ error$$

$$D_{kl} : Kull - back\ Divergence$$

In the proposed model, a total of nine residual blocks which contain two one-dimensional CNN layers, two batch normalization layers, and a ReLU activation function were used in the encoder, and the stride was set at 1. The size of latent variable Z was set at 60. The decoder was set to appear symmetrically with the encoder.
