# Supplementary material for: Unsupervised feature learning for electrocardiogram data using the convolutional variational autoencoder
Source: PLoS One. 2021 Dec 1;16(12):e0260612. doi: 10.1371/journal.pone.0260612 (PMC8635334; doi:10.1371/journal.pone.0260612)
Supplement: S2 Appendix — (PDF) [file pone.0260612.s002.pdf]

S2 Appendix. Assumption of anomaly detection

Reconstruction error as anomality score

We performed anomaly detection in electrocardiograms (ECGs) using reconstruction error as the abnormality score. As reconstruction error is dependent on the latent feature variables, anomaly detection is another way to represent the usefulness of feature variables. Two assumptions are made for anomaly detection using reconstruction error. First, there are many normal waveforms in naturally occurring large-scale data, and the number of ECGs with unusual arrhythmia or noise data will be smaller than that of those with normal waveforms. Therefore, it can be expected that the convolutional variational autoencoder (CVAE) will have a low reconstruction error for normal ECGs, and conversely, the reconstruction error will be high for arrhythmias or noise data. Second, the reconstruction errors may be interpreted with confidence if the Kull-back divergence is converged. According to the CVAE optimization equation below, the reconstruction error of data X is inversely proportional to the lower bound of the probability that actual X exists.

$$\log(P(X) \geq -Reconstruction\ Error(X, X') - KLD(N(\mu, \Sigma)|N(0, I))$$

$$P(X) \geq e^{-Reconstruction\ Error(X,X')}, assuming\ KLD\ is\ converged$$

$$P(X)\text{: } the\ probability\ of\ X\ in\ real\ world$$

$$KLD\text{: } Kull - back\ divergence$$

For example, if the lower bound of P(X) is 0.9, the probability of the occurrence of X is between 0.9 and 1.0, and it can be trusted that X is a waveform that often occurs in the actual distribution. However, if the lower bound of P(X) is 0.1, the probability of occurrence of X is between 0.1 and 1.0. X is not likely to occur frequently in the actual distribution and such data regarded as abnormal electrocardiograms in this study.
