# Supplementary material for: Unsupervised feature learning for electrocardiogram data using the convolutional variational autoencoder
Source: PLoS One. 2021 Dec 1;16(12):e0260612. doi: 10.1371/journal.pone.0260612 (PMC8635334; doi:10.1371/journal.pone.0260612)
Supplement: S3 Appendix — (PDF) [file pone.0260612.s003.pdf]

S3 Appendix. Anomaly detection comparison with long short-term memory (LSTM) Variational autoencoder (VAE)

To compare the anomaly detection performance of our model with the previous VAE-based anomaly detection model, we performed an additional anomaly detection experiment using LSTM-VAE as in a previous study. Long short-term memory (LSTM) is a deep-learning architecture that contains special structures to keep long-term and short-term memory based on a recursive recurrent neural network created to analyze time series data. LSTM-VAE has the same VAE structure as our model, but LSTM-VAE used LSTM for encoder and decoder. Additionally, in the previous study, only 1 second ECG data were studied, but we utilized about 8 seconds ECG data in the present study. We set the same model and structure to anomaly detection as in the previous study, but only the length of the ECG data was different. To compare extraction efficiency, the feature vector size was set as 60, which is similar to that in our study.

The figure below shows a reconstruction example of LSTM-VAE. It can be seen that only the last 1 second of ECG can be restored roughly, but the previous ECG shape cannot be reconstructed at all. LSTM-based models have limitations as the longer the input, the more difficult it is to learn data. The results were consistent with the limitation of LSTM. Moreover, we found that suggested CVAE was more suitable than LSTM-VAE when VAE was trained with ECGs for more than 8 seconds at 250 Hz. Additionally, anomaly detection was performed through reconstruction error in the same way as in our study. The table below shows the performance measures as the threshold is changed and the results of applying the threshold. In the Shaoxing dataset and MIT-BIT, f1-scores of LSTM-VAE were 0.60 and 0.34, respectively, which were worse than those in our model (CVAE) by 0.22 and 0.39, respectively. Additionally, to compare complexity of two models, we measured the number of ECGs processed per second. CVAE and LSTM-VAE dealt with 47.6 and 4.74 ECGs per second, respectively. This indicates

that CVAE is about 10 times more computationally efficient than LSTM-VAE.

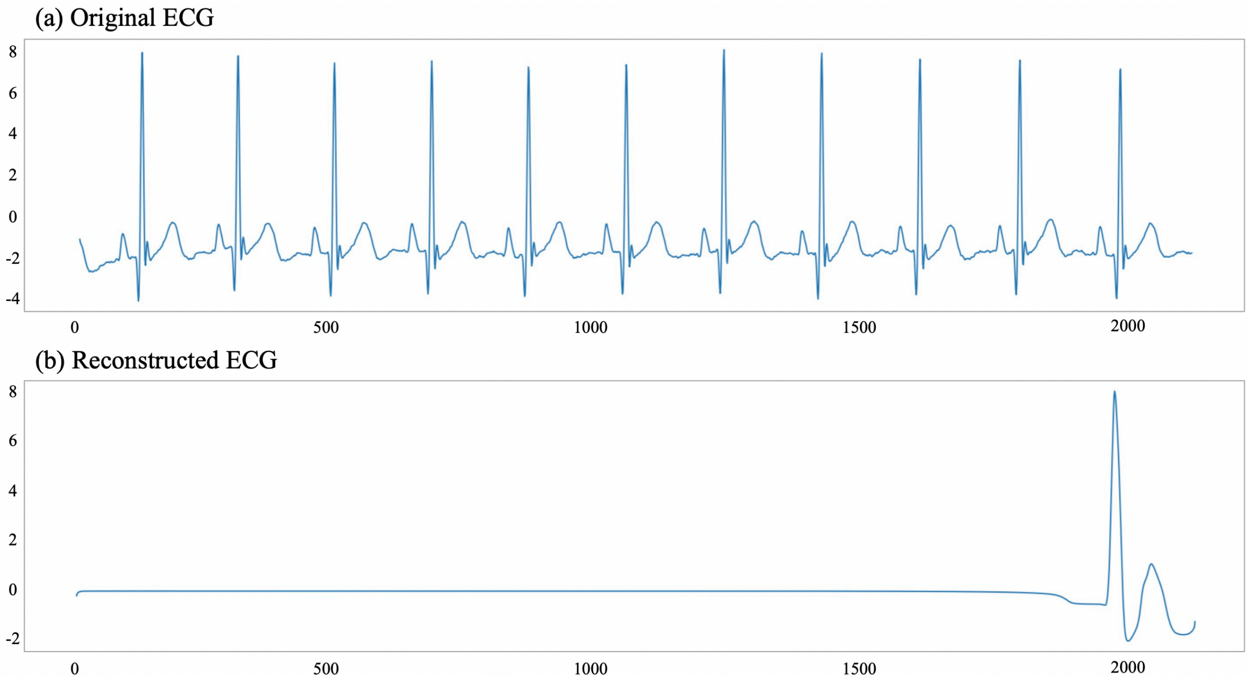

Reconstruction example of long short-term memory (LSTM)-variational autoencoder (VAE) (a) original ECG data (b) result of reconstructed ECG as LSTM-VAE output. End of 1 s ECG only can be roughly reconstructed.

Anomaly detection in the Shaoxing and MIT-BIH datasets

**Dataset**	**Threshold**	**Weighted f1-score**	**Accuracy**	**Normal**		**Abnormal**	
				Precision	Recall	Precision	Recall
**Shaoxing**	0.45	0.12	0.28	0.83	0.00	0.28	0.99
	0.55	0.13	0.28	0.82	0.00	0.28	0.99
	0.65	0.14	0.28	0.74	0.01	0.28	0.99
	0.75	0.15	0.29	0.68	0.02	0.28	0.97
	0.85	0.18	0.30	0.69	0.04	0.29	0.95
	0.95[a]	0.60	0.72	0.72	0.99	0.33	0.01
**MIT-BIH**	0.95	0.33	0.51	0.51	1.00	0.00	0.01

[a]The threshold with the highest micro f1-score was selected as the criteria for abnormal electrocardiograms.
