# Supplementary material for: Unsupervised feature learning for electrocardiogram data using the convolutional variational autoencoder
Source: PLoS One. 2021 Dec 1;16(12):e0260612. doi: 10.1371/journal.pone.0260612 (PMC8635334; doi:10.1371/journal.pone.0260612)
Supplement: S1 Fig — (PDF) [file pone.0260612.s004.pdf]

S1 Figure. Visualization of T-SNE and LLE clustering

The results of t-stochastic neighbor embedding (T-SNE) and locally linear embedding (LLE) clustering with normal sinus rhythms and each arrhythmia are shown. Clustering is not observed for Sinus irregularity (SI) and sinus atrium to atrial wander rhythms (SAAWR). As SI is also in the normal group according to an original paper of Shaoxing dataset, SI has no clustered group. The number of SAAWR samples was too small to determine the characteristics of SAAWR. AF: atrial flutter; AT: atrial tachycardia; AFIB: atrial fibrillation; AVRT: atrioventricular reentrant tachycardia; SB: sinus bradycardia; SR: sinus rhythm; ST: sinus tachycardia; SVT: supraventricular tachycardia; T-SNE: t-stochastic neighbor embedding

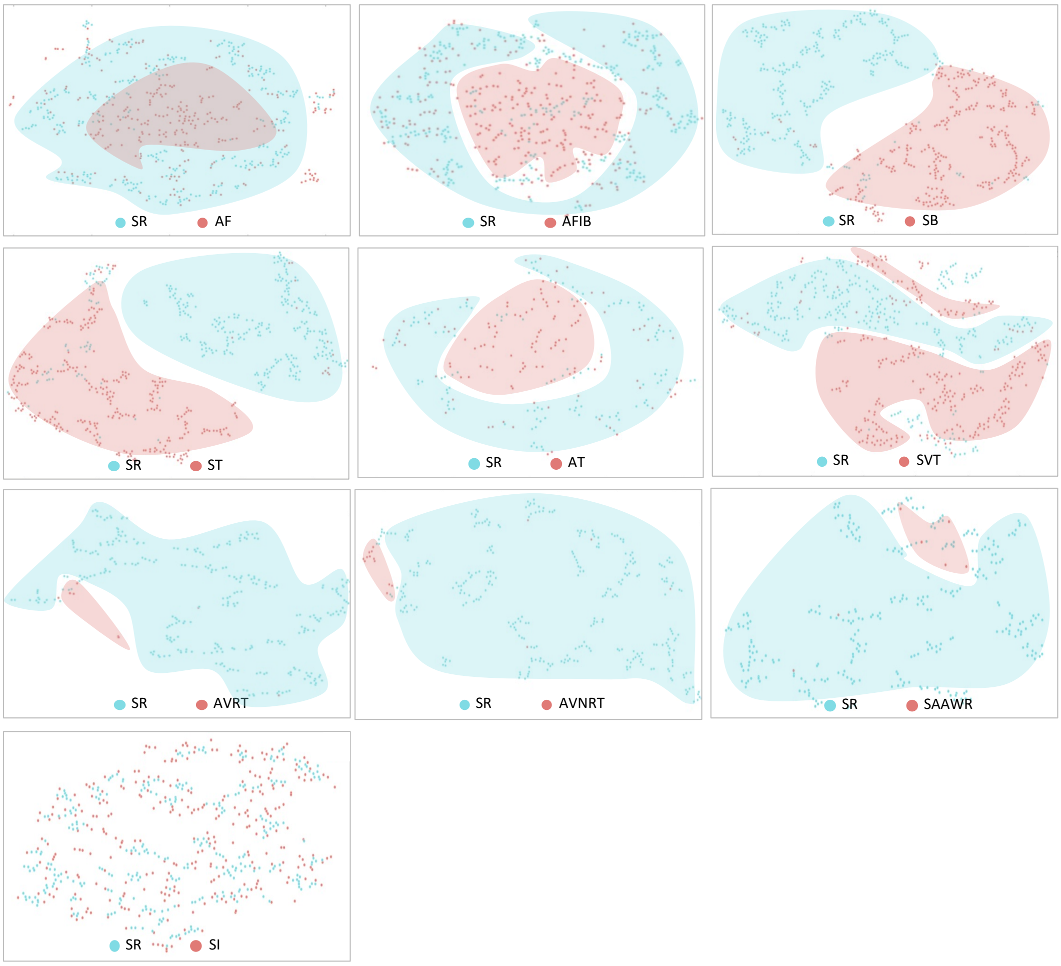

(A) Results of T-SNE for each arrhythmia

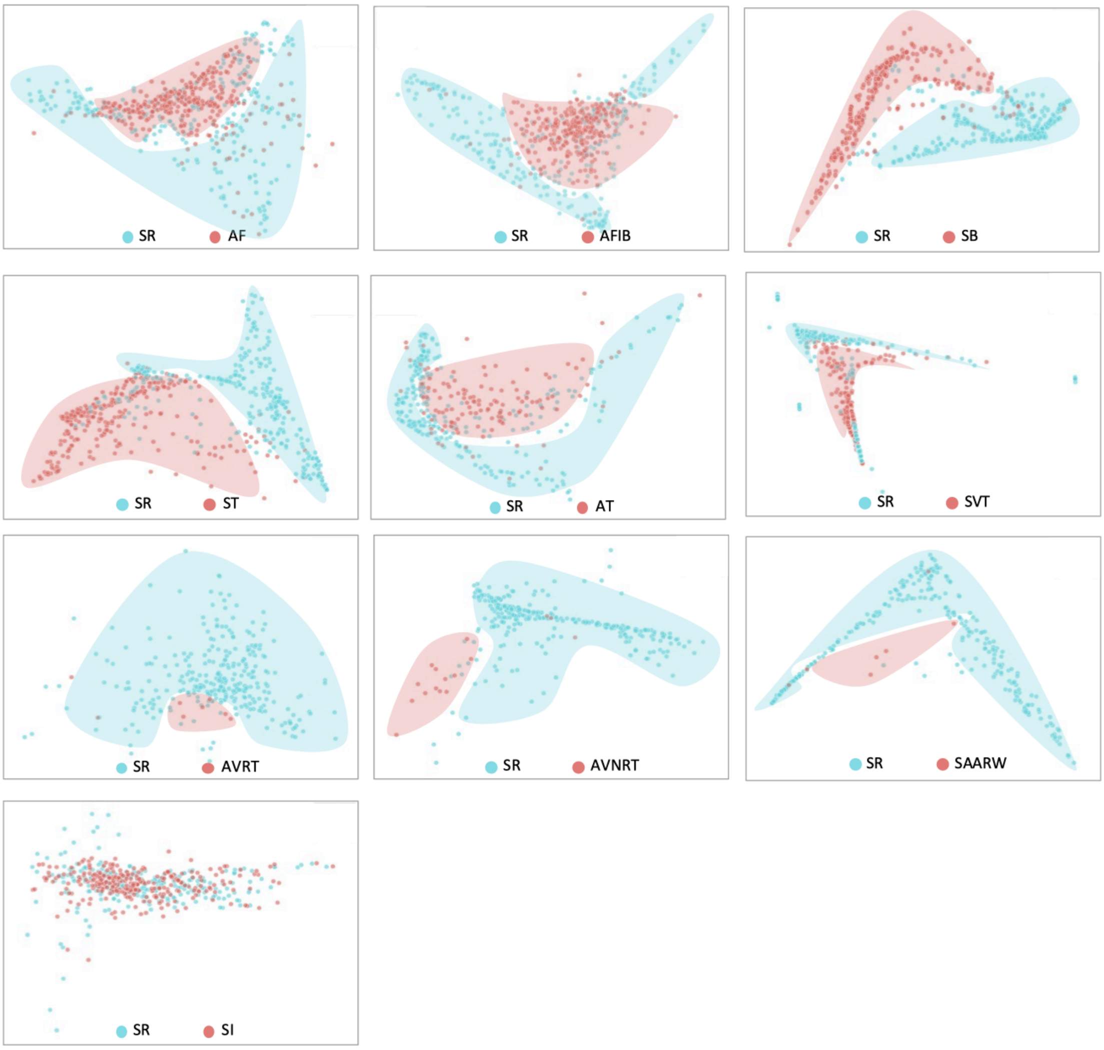

(B) Results of LLE for each arrhythmia
