# Supplementary material for: Unsupervised feature learning for electrocardiogram data using the convolutional variational autoencoder
Source: PLoS One. 2021 Dec 1;16(12):e0260612. doi: 10.1371/journal.pone.0260612 (PMC8635334; doi:10.1371/journal.pone.0260612)

S2 Figure. Performance of the classification of electrocardiograms into 11 rhythms in a validation dataset for each epoch

The weighted average of f1-scores, precision, and recall for transfer learning and random initialization are shown. The results of transfer learning (orange) and random initialization (blue) are shown. Transfer learning reached the peak point earlier than random initialization in the overall metrics.

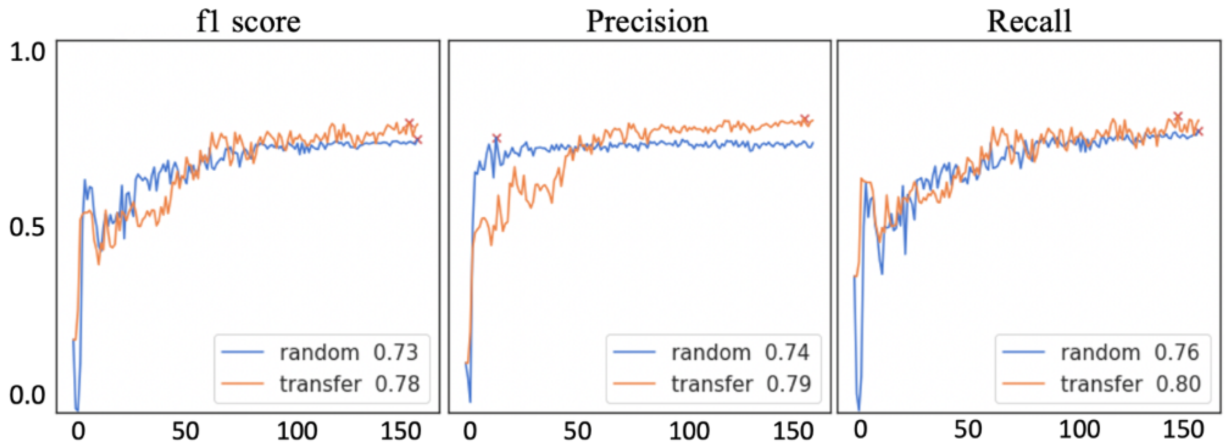

Supplement: S2 Fig — (PDF) [file pone.0260612.s005.pdf]
