# Supplementary material for: Unsupervised feature learning for electrocardiogram data using the convolutional variational autoencoder
Source: PLoS One. 2021 Dec 1;16(12):e0260612. doi: 10.1371/journal.pone.0260612 (PMC8635334; doi:10.1371/journal.pone.0260612)

S3 Figure. Structure of transfer learning of weight initialization

It shows the structure of transfer learning. The weights of the CVAE were used for weight initialization of arrhythmia classification.

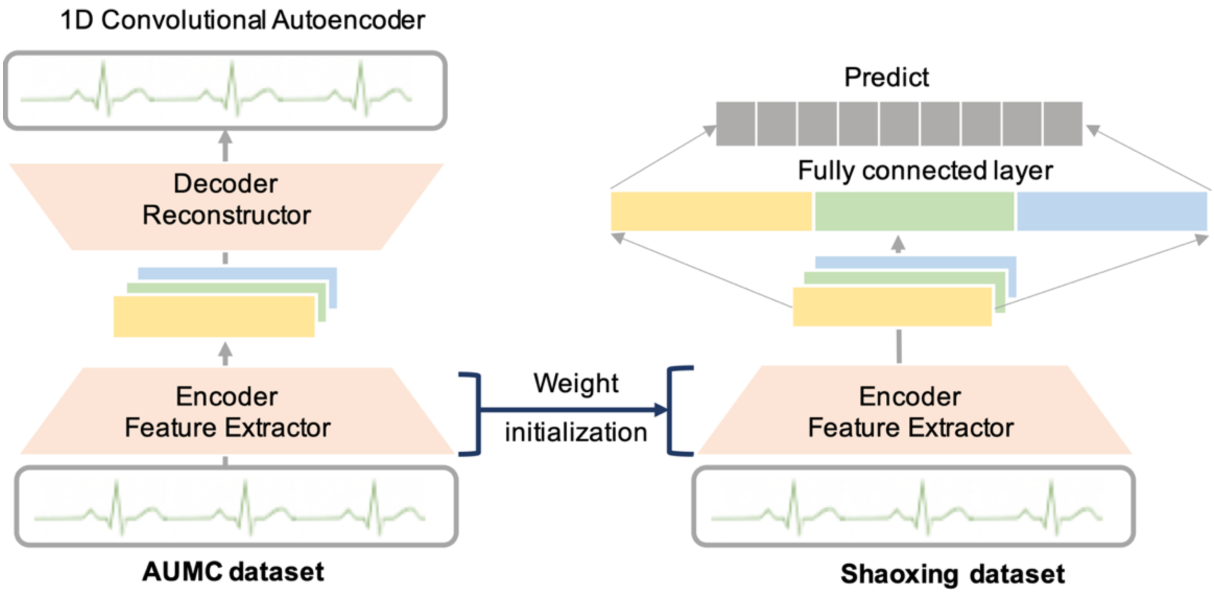

Supplement: S3 Fig — (PDF) [file pone.0260612.s006.pdf]
