# Supplementary material for: Unsupervised feature learning for electrocardiogram data using the convolutional variational autoencoder
Source: PLoS One. 2021 Dec 1;16(12):e0260612. doi: 10.1371/journal.pone.0260612 (PMC8635334; doi:10.1371/journal.pone.0260612)
Supplement: S1 Table — (PDF) [file pone.0260612.s007.pdf]

S1 Table. Detailed results of classification of 11 rhythms using XGBoost

We conducted arrhythmia classification with different combinations of feature sets to show that the features generated by CVAE and conventional features were mutually complimentary. The evaluation result of arrhythmia less than 30 samples was zero for every validation.

		Validation dataset					
		CVAE features			CVAE + anomality features		
	N	f1-score Mean ([a]std)	Precision Mean (std)	Recall Mean (std)	f1-score Mean (std)	Precision Mean (std)	Recall Mean (std)
Sinus Bradycardia	777	0.96 (0.00)	0.97 (0.00)	0.94 (0.00)	0.96 (0.01)	0.97 (0.01)	0.94 (0.01)
Sinus Rhythm	365	0.86 (0.02)	0.91 (0.04)	0.81 (0.01)	0.87 (0.02)	0.91 (0.05)	0.84 (0.01)
Atrial Fibrillation	356	0.74 (0.02)	0.82 (0.02)	0.67 (0.02)	0.76 (0.01)	0.87 (0.01)	0.68 (0.02)
Sinus Tachycardia	313	0.86 (0.02)	0.87 (0.04)	0.81 (0.01)	0.87 (0.01)	0.88 (0.02)	0.87 (0.01)
Supraventricular Tachycardia	117	0.07 (0.04)	0.04 (0.02)	0.21 (0.09)	0.08 (0.03)	0.05 (0.01)	0.24 (0.08)
Atrial Flutter	89	0.11 (0.04)	0.07 (0.03)	0.47 (0.13)	0.16 (0.07)	0.10 (0.05)	0.53 (0.16)
Sinus Irregularity	79	0.75 (0.03)	0.77 (0.06)	0.72 (0.02)	0.77 (0.03)	0.79 (0.05)	0.75 (0.03)
Atrial Tachycardia	24	0.00 (0.00)	0.00 (0.00)	0.00 (0.00)	0.00 (0.00)	0.00 (0.00)	0.00 (0.00)
Atrioventricular Node Reentrant Tachycardia	3	0.00 (0.00)	0.00 (0.00)	0.00 (0.00)	0.00 (0.00)	0.00 (0.00)	0.00 (0.00)
Sinus Atrium to Atrial Wandering Rhythm	1	0.00 (0.00)	0.00 (0.00)	0.00 (0.00)	0.00 (0.00)	0.00 (0.00)	0.00 (0.00)
Atrioventricular Reentrant Tachycardia	1	0.00 (0.00)	0.00 (0.00)	0.00 (0.00)	0.00 (0.00)	0.00 (0.00)	0.00 (0.00)

[a]std : standard deviation
