# Supplementary material for: Unsupervised feature learning for electrocardiogram data using the convolutional variational autoencoder
Source: PLoS One. 2021 Dec 1;16(12):e0260612. doi: 10.1371/journal.pone.0260612 (PMC8635334; doi:10.1371/journal.pone.0260612)
Supplement: S2 Table — (PDF) [file pone.0260612.s008.pdf]

S2 Table. Feature importance of XGBoost

Table shows the list of features in order of importance for classifying arrhythmia using XGboost.

CVAE features		CVAE + anomality features	
Feature name	Importance	Feature name	Importance
8th	0.04	Anomality score	0.50
58th	0.04	9th	0.18
9th	0.03	8th	0.08
48th	0.03	58th	0.04
47th	0.03	7th	0.03
52th	0.03	48th	0.03
15th	0.02	31th	0.02
7th	0.02	15th	0.02
31th	0.02	52th	0.02
57th	0.02	57th	0.02
