# Supplementary material for: Unsupervised feature learning for electrocardiogram data using the convolutional variational autoencoder
Source: PLoS One. 2021 Dec 1;16(12):e0260612. doi: 10.1371/journal.pone.0260612 (PMC8635334; doi:10.1371/journal.pone.0260612)
Supplement: S3 Table — (PDF) [file pone.0260612.s009.pdf]

S3 Table. Classification of arrhythmias in the Shaoxing dataset using weight initialization of transfer learning

Name	Validation set						
		Random initialization[a]			Transfer learning[a]		
	N	f1-score	Precision	Recall	f1-score	Precision	Recall
Sinus Bradycardia	778	0.97 (0.00)	0.96 (0.01)	0.99 (0.00)	0.93 (0.01)	0.96 (0.04)	0.92 (0.06)
Sinus Rhythm	370	0.71 (0.40)	0.71 (0.40)	0.72 (0.40)	0.84 (0.04)	0.85 (0.05)	0.85 (0.09)
Atrial Fibrillation	357	0.53 (0.48)	0.51 (0.47)	0.55 (0.50)	0.71 (0.18)	0.78 (0.05)	0.70 (0.26)
Sinus Tachycardia	329	0.94 (0.01)	0.94 (0.02)	0.93 (0.02)	0.91 (0.02)	0.92 (0.06)	0.90 (0.04)
Supraventricular Tachycardia	102	0.80 (0.04)	0.77 (0.05)	0.85 (0.04)	0.49 (0.28)	0.40 (0.27)	0.69 (0.41)
Atrial Flutter	88	0.41 (0.07)	0.37 (0.15)	0.61 (0.19)	0.25 (0.06)	0.35 (0.14)	0.24 (0.12)
Sinus Irregularity	75	0.51 (0.15)	0.64 (0.31)	0.56 (0.21)	0.36 (0.15)	0.48 (0.16)	0.37 (0.32)
Atrial Tachycardia	23	0.15 (0.13)	0.20 (0.16)	0.21 (0.19)	0.06 (0.08)	0.06 (0.09)	0.05 (0.07)
Atrioventricular Node Reentrant Tachycardia	6	0.17 (0.26)	0.19 (0.32)	0.20 (0.27)	0.04 (0.1)	0.03 (0.07)	0.06 (0.15)
Sinus Atrium to Atrial Wandering Rhythm	0	-	-	-	-	-	-
Atrioventricular Reentrant Tachycardia	0	-	-	-	-	-	-

[a]Results at the Nth epoch where the model showed the best weighted f1-score for 150 epochs.
